# Does Thymoma Plus Hypogammaglobulinemia Define Good Syndrome? A 30-Year Longitudinal Study of a Single Patient with Persistent B-Cell Preservation

**DOI:** 10.3390/ijms27219726

**Published:** 2026-10-29

**Authors:** Daniel Iguasnia Portilla, Isabel Castillo Castillo, Valeria Franco Figueroa, Helena Fernández Cabrera, Silvia Romero Chala, Irina Sobieschi, Hector Cordero, Luis Miguel Fernández Pereira

**Affiliations:** 1 Department Service of Immunology and Genetics, Hospital Universitario San Pedro de Alcántara, 10003 Cáceres, Spain; 2 Immunology Unit, Department of Physiology, Faculty of Veterinary Medicine, Universidad de Extremadura, 10003 Cáceres, Spain

**Keywords:** Good syndrome, thymoma, hypogammaglobulinemia, antibody deficiency, B cells, immunophenotyping, thymoma-associated immunodeficiency, immune phenocopy

## Abstract

Good syndrome is commonly diagnosed when thymoma and hypogammaglobulinemia coexist, although universally accepted diagnostic criteria are lacking. Profound B-cell lymphopenia is one of its most consistent immunological features. We describe a woman followed for 30 years in our unit whose severe antibody deficiency preceded thymoma detection by 14 years. Circulating B cells were preserved before thymectomy and throughout 16 years of subsequent follow-up, with maintained class-switched memory B cells despite persistent antibody deficiency. Her clinical course was favorable under immunoglobulin replacement, without documented major opportunistic infections. T-cell differentiation abnormalities were present despite preserved absolute counts. These findings suggest that patients currently classified as Good syndrome may represent a more heterogeneous group than traditionally assumed. Although preserved B cells do not exclude Good syndrome under existing definitions, the present observation should be considered hypothesis-generating and supports further studies evaluating potential biological heterogeneity among patients diagnosed with Good syndrome. This case highlights the need for consensus diagnostic criteria that consider the temporal relationship between both conditions, B-cell numbers, and the accompanying immune abnormalities, rather than relying on the coexistence of thymoma and hypogammaglobulinemia alone.

## 1. Introduction

Good syndrome (GS) describes the association of thymoma and hypogammaglobulinemia, reported in detail by Good and colleagues in 1956 [1]. These original observations, together with Good’s subsequent investigations, contributed fundamentally to the recognition of the thymus as a central organ of the immune system and helped define the distinct contributions of thymus-dependent and antibody-producing lymphoid lineages to cellular and humoral immunity. The diagnosis usually rests on the coexistence of these findings, although universally accepted diagnostic criteria are lacking. The commonly described immune phenotype includes markedly reduced or absent circulating B cells, variable CD4+ T-cell lymphopenia, an inverted CD4/CD8 ratio, impaired cellular immunity, opportunistic infections, and autoimmune or hematological complications [2,3].

Profound B-cell depletion is consistently reported in recent cohorts. Absolute B-cell counts were very low in all 78 patients in the United Kingdom registry series [4]. A systematic review of 162 individually reported cases found absent or reduced B cells in 118 of the 124 patients with available data (95.2%); six (4.8%) had normal counts [5]. Detailed flow cytometry identified reduced B cells in all nine patients in another study, with fewer than 0.1 cells/µL in eight [6]. A 2026 study also documented absence of B cells in blood and sampled lymphoid tissues, alongside cellular immune abnormalities and anti-cytokine antibodies [7]. B-cell depletion is therefore a characteristic finding, but preserved B cells are a recognized exception.

Current clinical definitions do not require B-cell depletion. The 2019 European Society for Immunodeficiencies (ESID) working definition for thymoma with immunodeficiency requires thymoma and serum IgG more than two standard deviations below the age-adjusted mean, without a B-cell threshold [8]. The 2025 AAAAI/ACAAI/CIS practice parameter states that patients with hypogammaglobulinemia and thymoma should be given a diagnosis of GS [9]. The 2024 International Union of Immunological Societies (IUIS) classification lists GS among phenocopies of inborn errors of immunity (IEI) and describes decreased CD4+ T cells, increased CD8+ T cells, absent B cells, reduced immunoglobulins, and invasive or opportunistic infections as characteristic features [10]. These documents serve different purposes, but they leave uncertainty about which immune findings should carry diagnostic weight.

This raises a clinically important question: should a thymoma detected years later automatically lead to the reclassification of a pre-existing antibody deficiency as GS? Studies have identified impaired early B-cell development in GS [11], while the broader term thymoma-associated immunodeficiency (TAI) includes abnormalities beyond the classical syndrome [12,13,14].

We describe 30 years of clinical follow-up, including approximately 16 years of documented B-cell phenotyping, in a patient whose severe antibody deficiency preceded thymoma detection by 14 years. The persistent preservation of B cells before and after thymectomy provides an opportunity to examine the limits of the current definition and the need for diagnostic consensus.

## 2. Results

### 2.1. Severe Antibody Deficiency Preceded Thymoma Detection by 14 Years

The patient had an unremarkable perinatal and early childhood history apart from measles at three years of age. She reported occasional upper respiratory infections without hospital admission or school absence, recurrent herpes labialis during adolescence and young adulthood, and persistent loose stools from adolescence. In 1996, at 33 years of age, she developed vomiting, profuse diarrhea, and a 16 kg weight loss. Laboratory evaluation showed severe panhypogammaglobulinemia (IgG < 148 mg/dL, IgA < 25.9 mg/dL, and IgM < 19 mg/dL). Before initiation of immunoglobulin replacement therapy, stool cultures, parasitological studies, and investigations for opportunistic infectious agents, including cytomegalovirus, were performed. Giardia lamblia cysts were identified in stool, whereas no other enteric pathogens or opportunistic infections were detected. Therefore, intravenous immunoglobulin replacement was started, followed by clinical improvement, although antibody deficiency persisted and was documented during the follow-ups even after the eradication of Giardia and the resolution of the acute gastrointestinal episode. After approximately nine years of immunoglobulin replacement, the patient was referred to our immunology department, precluding assessment of baseline vaccine responses.

Gastrointestinal symptoms dominated the early course. At 35, she was admitted with recurrent gastroenterocolitis and weight loss; stool cultures and parasitology were negative. Chronic loose stools gradually resolved. At 42, upper gastrointestinal evaluation showed chronic atrophic gastritis with intestinal metaplasia, *Helicobacter pylori* infection, and nodular duodenitis; eradication required three antibiotic regimens. At 46, capsule endoscopy was normal.

At 44 years old, high-resolution chest computed tomography (HRCT) was described as normal in the clinical record, without a detectable thymic mass, bronchiectasis, or parenchymal disease. At 47, CT identified a small anterior mediastinal lesion, subsequently confirmed as thymoma. Thymectomy was performed and adjuvant radiotherapy was initiated. Figure 1 and Table 1 summarize the clinical course.

The infectious course under immunoglobulin replacement was relatively favorable. No mucocutaneous candidiasis, *Pneumocystis jirovecii* pneumonia, cytomegalovirus disease, or other major opportunistic infections were documented. Myasthenia gravis, lichen planus, pure red-cell aplasia, and autoimmune cytopenias were not recorded. Hypothyroidism, atrophic gastritis, and iron-deficiency anemia were additional clinical features.

### 2.2. B Cells Remained Preserved Before and After Thymectomy

At age 47, before diagnostic biopsy and thymectomy, CD19+ B cells accounted for 21.5% of lymphocytes, with an absolute count of 658 cells/µL. Thoracic CT performed earlier had already identified the lesion, documenting preserved B cells in the presence of radiologically detectable thymoma. Subsequent measurements showed continued B-cell preservation (Figure 2; Supplementary Table S1).

The longitudinal series comprises 23 CD19+ observations, including preoperative measurement. When she was 50 years old, one record was excluded because its percentage (22.1%) and absolute count (130 cells/µL) were inconsistent with the accompanying total lymphocyte count. From January 2016 through September 2026, 20 measurements ranged from 236 to 667 cells/µL, with a median of 435.5 cells/µL.

At 63, CD19+ B cells were 599 cells/µL (21.85%), slightly above the laboratory reference intervals of 100–500 cells/µL and 6–19%. Light-chain expression was polyclonal, with κ and λ expression in 59% and 41% of B cells, respectively.

### 2.3. Preserved Class-Switched Memory B Cells and Expanded CD21-Low Cells

Eight B-cell subset assessments of the patient from November 2017 through July 2022, at ages 54 to 59, showed class-switched memory cells (CD27+IgD−IgM−) at 10–16% of CD19+ B cells (median, 15%) and CD21-low/negative cells at 19–39% (median, 25%). During the last determination performed at 63, both populations represented 15% of B cells (Figure 3; Table 2). The switched memory fraction was within the laboratory interval of 8–23%, whereas CD21-low cells remained above the interval of 0–10%. Total CD27+ memory B cells accounted for 46% of B cells (reference interval, 15–35%). The reported CD38+IgM+ and CD38+IgM− populations were 2.5% and 0%, respectively (Table 3; Supplementary Table S3). Class-switched memory proportions remained above the 2% threshold used to identify severely reduced switched memory cells in the EURO class framework for common variable immunodeficiency (CVID) [15].

### 2.4. Thoracic Imaging and Thymoma Histopathology

In 2010, at 47, the thoracic CT showed an anterior mediastinal lesion (Figure 4), described in the imaging record as a lobulated mass measuring approximately 1.7 × 1.6 × 1.7 cm, with adjacent fat changes and anterior pericardial thickening, without pleural or pericardial effusion. PET-CT suggested metabolically indolent disease. Biopsy in July 2010 showed invasive WHO type B2 thymoma extending beyond the capsule into adjacent fat. The resection specimen obtained in September 2010 contained microscopic residual WHO type AB thymoma, with epithelial-predominant areas and no lymph-node metastases. The tumor was reported as stage II.

### 2.5. T- and NK-Cell Counts and T-Cell Differentiation

Pre-thymectomy CD4+, CD8+, and NK-cell counts were 1129, 797, and 211 cells/µL, respectively. Between November 2020 and September 2026, 14 assessments showed CD4+ counts of 670–1060, CD8+ counts of 546–1269, and NK-cell counts of 154–453 cells/µL, with CD4/CD8 ratios of 0.83–1.33. The ratio was intermittently inverted, without sustained profound CD4+ or NK-cell depletion (Supplementary Table S2 and Figure S1). An isolated routine blood count in August 2022 showed 0.85 × 10^9^ lymphocytes/L, without a corresponding subset analysis.

Major lymphocyte counts remained within their laboratory reference intervals in September 2026, except for the slightly elevated B-cell count (Table 3). CD45RA+CD27+ cells represented 7.06% of CD4+ and 3.55% of CD8+ cells and were reported as reduced. CD45RA+CD27− cells accounted for 63.32% of CD8+ cells. The full distributions are shown in Table 3 and Figure 5.

Phenotypically defined recent thymic emigrants (CD3+CD4+CD45RA+CD31+) accounted for 2.7% of total CD4+ cells, below the laboratory reference value of >7%.

### 2.6. Persistent Antibody Deficiency During Immunoglobulin Replacement

Serum IgG concentrations obtained after treatment began in 1996 (33 y/o) reflect immunoglobulin replacement and cannot be used to quantify endogenous IgG production. IgA and IgM remained very low, frequently below the reporting limit. Longitudinal concentrations and reporting limits are provided in Supplementary Table S4.

## 3. Discussion

Severe antibody deficiency preceded thymoma detection by 14 years, and B cells remained preserved before and after thymectomy. Although this patient meets commonly used definitions of GS [8,9], her course differs from the B-cell-depleted phenotype described in most cohorts [4,5,6,7]. These findings raise the question of whether patients with thymoma and hypogammaglobulinemia could share the same underlying biological mechanism. However, larger cohorts of patients are needed to help establish a definitive statement on the pathophysiology of the disease, since a single patient’s follow-up experience does not necessarily establish causality.

### 3.1. Current Definitions and the Need for Consensus

Early reviews explicitly noted the absence of formal GS diagnostic criteria [2,3]. As summarized in Table 4, currently available definitions and classification proposals for GS differ substantially regarding the weight assigned to specific clinical and immunological features. While all definitions require the coexistence of thymoma and hypogammaglobulinemia, considerable disagreement persists regarding the relevance of additional findings such as peripheral B-cell depletion, CD4 lymphopenia, an inverted CD4/CD8 ratio, or opportunistic infections. Current ESID and AAAAI/ACAAI/CIS guidance still define the condition through thymoma and hypogammaglobulinemia, without requiring a low B-cell count [8,9]. Selecting patients based on these two findings allows their clinical features to be described but does not establish that the antibody deficiency has the same cause in every patient.

IUIS places GS among IEI phenocopies and describes reduced CD4+ cells, increased CD8+ cells, and absent B cells as characteristic findings [10]. This classification summarizes the usual phenotype rather than setting validated diagnostic requirements; its accompanying text also recognizes presentations with normal or reduced B-cell counts. Neither the phenocopy designation nor the entry of “No B cells” establishes the cause of immunodeficiency in an individual patient. Neither the phenocopy designation nor the entry of “No B cells” establishes the cause of immunodeficiency in an individual patient. These discrepancies may explain why patients with markedly different immunological phenotypes continue to be grouped under the same diagnostic label. The present case illustrates this challenge, as the patient fulfills the core clinical criteria for GS despite persistent preservation of circulating B cells. Therefore, the differences between clinical definitions and phenotypic descriptions summarized in Table 4 support the need for consensus regarding which clinical and immunological features should be considered defining characteristics of GS and which should be regarded as associated, but non-mandatory, manifestations.

### 3.2. The Significance of Preserved B Cells

The consistency of severe B-cell depletion across cohorts [4,5,6,7] supports its potential diagnostic value. Kabir et al. proposed including reduced or absent B cells as an additional criterion [16]. A 2026 study of thymic epithelial tumors subsequently found that B-cell lymphopenia was consistently associated with GS across tumor-stage and autoimmune-disease groups [18]. However, cohort selection, differences in measurement, and patients with preserved B cells limit the use of a single count as an exclusion criterion.

Preserved B cells have been described in several patients with thymoma and hypogammaglobulinemia (Table 5). Manzanell et al. reported antibody deficiency developing 11 years after thymoma resection, with normal circulating B cells and marrow plasma cells but impaired T-helper function [19]. Montella et al. identified a patient with panhypogammaglobulinemia and a B-cell count within the study reference range; across their cohort, B-cell lymphopenia was more closely associated with recurrent infections than hypogammaglobulinemia [20]. Hashiro et al. described preserved B-cell counts in a patient with pure red-cell aplasia and subclinical myasthenia gravis [21]. Earlier functional observations by Brenner et al. also argued against a single mechanism for thymoma-associated hypogammaglobulinemia [22]. The present case therefore extends these observations through its long follow-up and the persistence of B cells despite antibody deficiency predating the tumor.

### 3.3. Disease Chronology and Causality

Hypogammaglobulinemia can precede thymoma detection. In the UKPID series, this occurred in nine of 78 patients, with intervals of 1–8 years [4]. In the historical systematic review, thymoma was diagnosed after infection or hypogammaglobulinemia in 26 of 132 evaluable patients, with intervals of up to 15 years [3]. Because that category combines infection and hypogammaglobulinemia, the longest interval does not necessarily represent 15 years of documented antibody deficiency. The 14-year interval in our patient exceeds the range in the UKPID cohort, and the intervening 2007 HRCT was described as showing no detectable thymic mass.

A normal earlier scan does not exclude microscopic thymic disease or an immune effect preceding the detectable tumor. Conversely, the later diagnosis of thymoma does not establish that it caused an antibody deficiency already present for many years. The chronology therefore warrants considering a pre-existing antibody disorder without proving that the two conditions were unrelated.

### 3.4. B-Cell Development and Proposed Mechanisms

Studies of B-cell development offer a possible explanation for the profound depletion seen in GS. Hayward et al. showed that T lymphocytes from thymoma patients with hypogammaglobulinemia suppressed allogeneic pre-B-cell proliferation in vitro [23]. Masci et al. identified oligoclonal bone-marrow CD8+ BV8 T-cell expansion in five thymoma patients with B-cell lymphopenia, but not in those with preserved B cells [24]. Del Pino Molina et al. found very low numbers of bone-marrow B-cell precursors and a differentiation arrest after the pro-B-cell stage [11].

These findings support a model in which disturbed thymic tolerance permits immune responses that interfere with early B-cell development. Altered AIRE expression and regulatory T-cell populations in thymomas [25], anti-cytokine autoantibodies [26], and the cellular abnormalities described in recent GS studies [7,27] provide further support. However, no single mechanism has been established for all patients diagnosed with GS, and anti-cytokine antibodies also occur in thymoma-associated immune disorders outside classical GS [12,26].

In addition, unlike monogenic inborn errors of immunity, no recurrent germline genetic defect has been consistently associated with Good syndrome [3,10]. Current evidence supports the concept that GS is predominantly an acquired disorder characterized by thymoma-associated immune dysregulation rather than a classical Mendelian disease. Nevertheless, the marked clinical and immunological heterogeneity reported among patients raises the possibility that genetic susceptibility factors or additional environmental triggers may contribute to disease development in selected cases.

Our patient did not develop the marked peripheral B-cell loss described in these studies. Nevertheless, preserved B-cell counts, polyclonal light-chain expression, and class-switched memory cells do not establish effective antibody secretion. Nor does the reported CD38+IgM− population demonstrate a terminal differentiation block, given the limited gating information and a reference interval that includes zero. Without functional studies, the site of the defect leading to antibody production remains undetermined.

The patient was initially classified as having CVID. Her B-cell findings overlap with some CVID phenotypes [15], but current criteria cannot be applied retrospectively with certainty because vaccine responses before immunoglobulin replacement are unavailable. A distinct underlying antibody disorder remains a possibility without requiring a definitive alternative diagnosis.

### 3.5. T-Cell Differentiation Abnormalities

Preserved total CD4+ and CD8+ counts coexisted with reduced CD45RA+CD27+ fractions, a large CD8+CD45RA+CD27− population and a reduced proportion of phenotypically defined recent thymic emigrants. These findings show that immune abnormalities were not confined to antibody production. They do not, however, establish functional T-cell impairment or identify its cause.

Adult thymectomy has been associated with reduced new T-cell production during long-term follow-up [28]. The T-cell findings must therefore be interpreted in the context of age, prior to thymectomy, radiotherapy, and underlying immune disorder. The CD31-based recent-thymic-emigrant measurement is an indirect phenotypic indicator rather than a direct measure of thymic output. These observations are insufficient to attribute the T-cell changes specifically to GS.

### 3.6. A Broader Spectrum of Thymoma-Associated Immunodeficiency

TAI has been used to include classical GS and other immune disturbances associated with thymoma [12,13]. Recent cohorts describe NK-cell abnormalities, B-cell lymphopenia, isolated immunoglobulin deficiencies, and other presentations beyond the usual thymoma–hypogammaglobulinemia combination [14,17].

The present patient may have pre-existing antibody deficiency followed by thymoma, an uncommon B-cell-preserved form of GS, or overlapping immune disorders. A single case cannot distinguish these possibilities. Nonetheless, the prolonged preservation of B cells argues against assuming a shared disease mechanism solely because thymoma and hypogammaglobulinemia coexist.

### 3.7. Clinical Implications and Limitations

Consensus criteria should address the temporal relationship between antibody deficiency and thymoma, the degree and persistence of B-cell depletion, and associated cellular and clinical findings. Until then, the association should prompt assessment of B-, T-, and NK-cell numbers and, when available, their maturation subsets. Prospective multicenter studies are needed to determine how these findings distinguish different forms of thymoma-associated immunodeficiency. Treatment and tumor surveillance should remain guided by clinical needs rather than by the diagnostic label alone.

Because this study describes a single patient, no causal relationships can be established, and no modification of existing diagnostic definitions can be proposed based on these observations alone. Rather, this report should be regarded as a hypothesis-generating study that highlights areas requiring further evaluation in larger multicentric cohorts. The strengths of this study are the long follow-up, antibody deficiency preceding thymoma, an intervening HRCT reported as normal, and repeated B-cell measurements before and after thymectomy. Its main limitations are the single-patient design and changes in laboratory methods over time. Immunoglobulin replacement also limits interpretation of subsequent IgG concentrations and bacterial infection frequency. The observations support reconsideration of diagnostic criteria but cannot establish a separate disease or a causal mechanism.

## 4. Materials and Methods

### 4.1. Study Design

We reviewed the clinical course and laboratory follow-up of this single patient from 1996 to September 2026 for the longitudinal observational study. Data comprised clinical history, serum immunoglobulins, lymphocyte immunophenotyping, thoracic imaging, and histopathology reports. This study was conducted in compliance with the ethical standards approved by the Ethics Committee of the Hospital San Pedro de Alcántara (IRB code 090-2026), and informed consent was obtained from the patient according to the principles of the Declaration of Helsinki.

### 4.2. Immunoglobulin Measurements

Serum IgG, IgA, and IgM were measured during routine care using an Optilite analyzer (The Binding Site, Birmingham, UK). IgG concentrations obtained during immunoglobulin replacement therapy (IgRT) were interpreted as measurements during treatment rather than as estimates of endogenous IgG synthesis.

### 4.3. Flow Cytometry

Flow cytometry was performed using a Navios flow cytometer (Beckman Coulter, Brea, CA, United States), and data were analyzed with Kaluza software Version 2.1 (Beckman Coulter). Absolute counts of CD3+, CD3+CD4+, CD3+CD8+, CD19+, and CD3−CD16+CD56+ cells were reported in cells/µL. B-cell subsets were described using CD27, IgD, IgM, CD21, and CD38, with proportions expressed as percentages of CD19+ B cells.

T-cell differentiation subsets were reported using CD45RA/CD45RO and CD27 combinations as percentages of CD4+ or CD8+ cells. The reported categories combined CD197-negative and -positive cells. Recent thymic emigrants were defined phenotypically as CD3+CD4+CD45RA+CD31+ cells and expressed as a percentage of total CD4+ cells.

### 4.4. Imaging and Histopathology

Coronal and axial CT images were available from the 2010 examination. Findings from the 2007 HRCT and the 2010 PET-CT were retrieved from the clinical record; the original 2007 images were not available for review. Histological subtype and stage were taken from the July and September 2010 pathology reports.

## 5. Conclusions

The coexistence of thymoma and hypogammaglobulinemia may encompass different disorders. In this patient, severe antibody deficiency preceded thymoma detection by 14 years, and circulating B cells remained preserved before and after thymectomy. This course differs from the profound B-cell depletion usually described in GS and raises the possibility of a distinct underlying process. Preserved B cells alone do not exclude the diagnosis, but the association of thymoma and hypogammaglobulinemia should not be assumed to establish a shared cause. Consensus diagnostic criteria are needed to clarify the role of disease chronology, B-cell depletion, and associated immune abnormalities in defining GS.

## Figures and Tables

**Figure 1:**
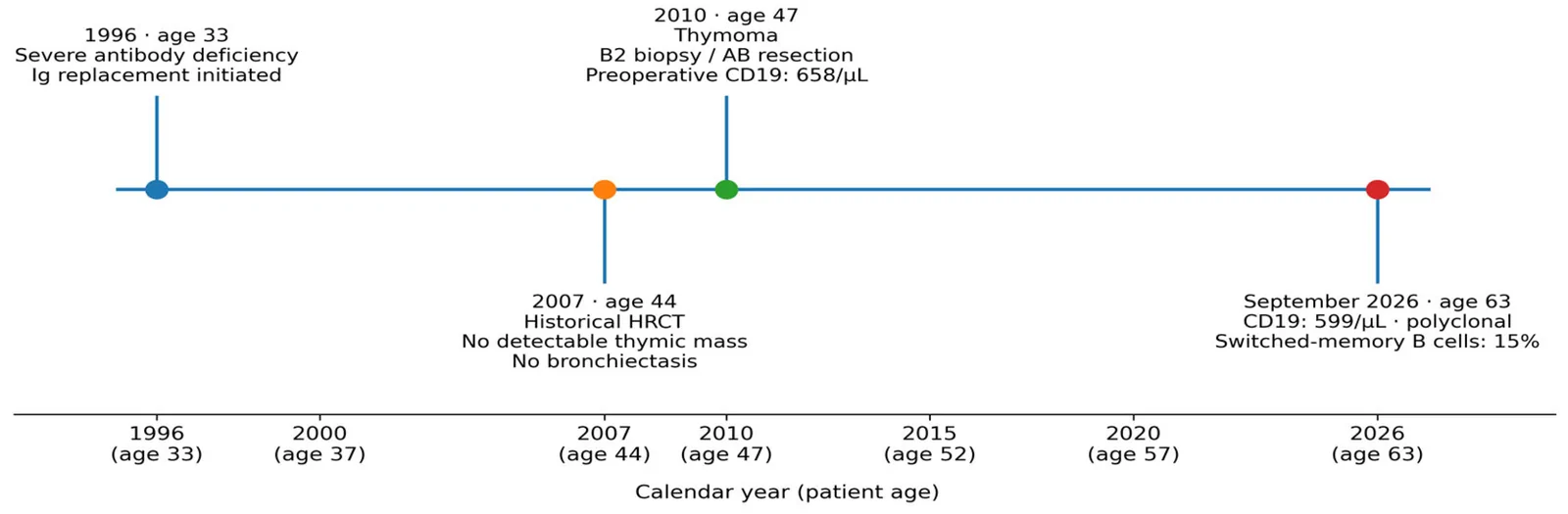
Clinical course, 1996–September 2026. Severe antibody deficiency preceded thymoma detection by 14 years. The 2007 HRCT findings are documented in the clinical record. The 2010 B-cell count was obtained before the thymectomy. The 2010 B-cell count was obtained before the thymectomy. Clinical follow-up spans approximately 30 years, with B-cell measurements available over approximately 16 years.

**Figure 2:**
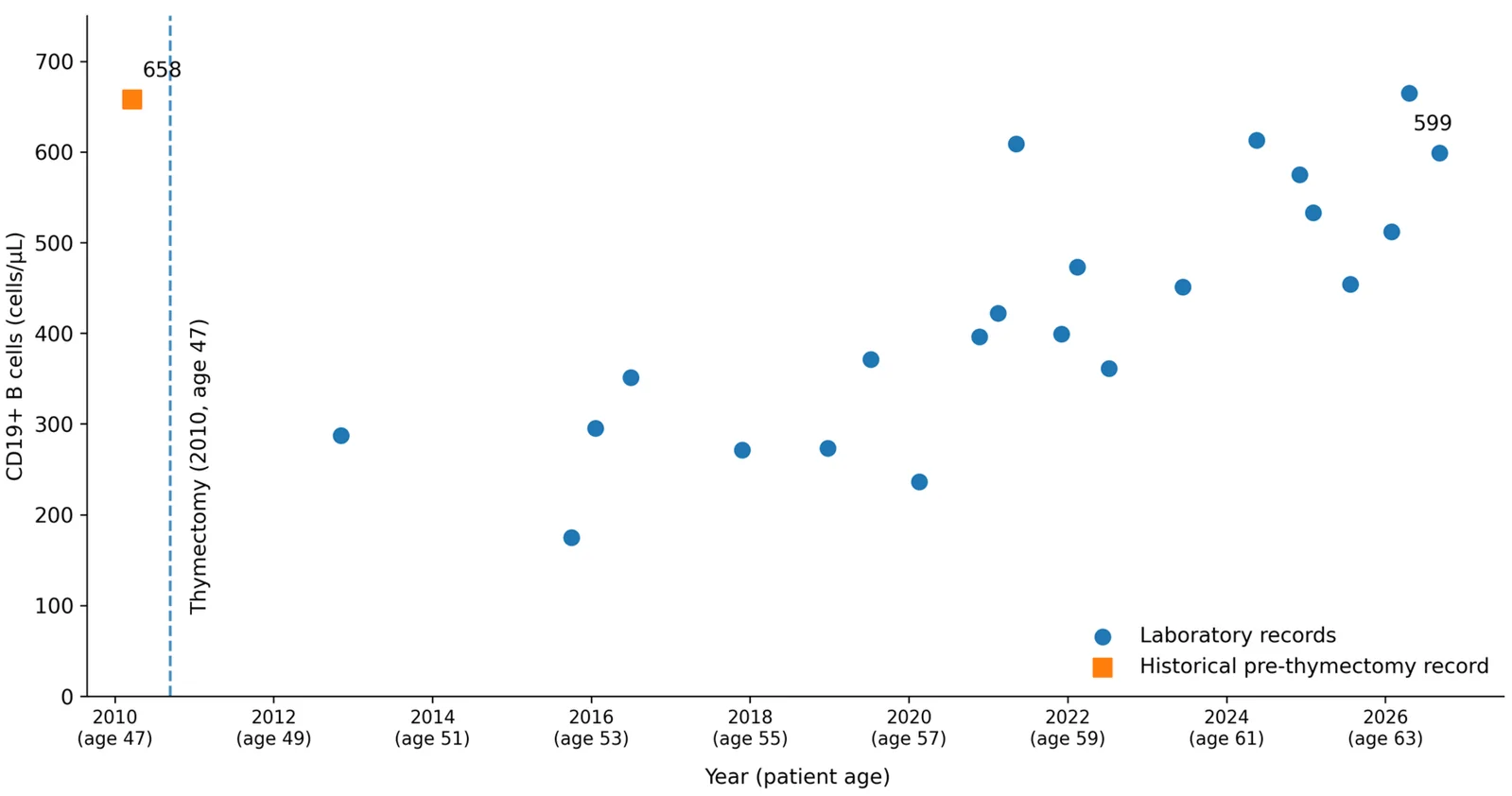
Absolute CD19+ B-cell counts, 2010–September 2026. Each symbol represents a dated observation. The square denotes the pre-thymectomy count, and circles denote subsequent laboratory measurements. The dashed line marks the thymectomy. The discordant August 2013 record is excluded; all records and their inclusion status are listed in Supplementary Table S1.

**Figure 3:**
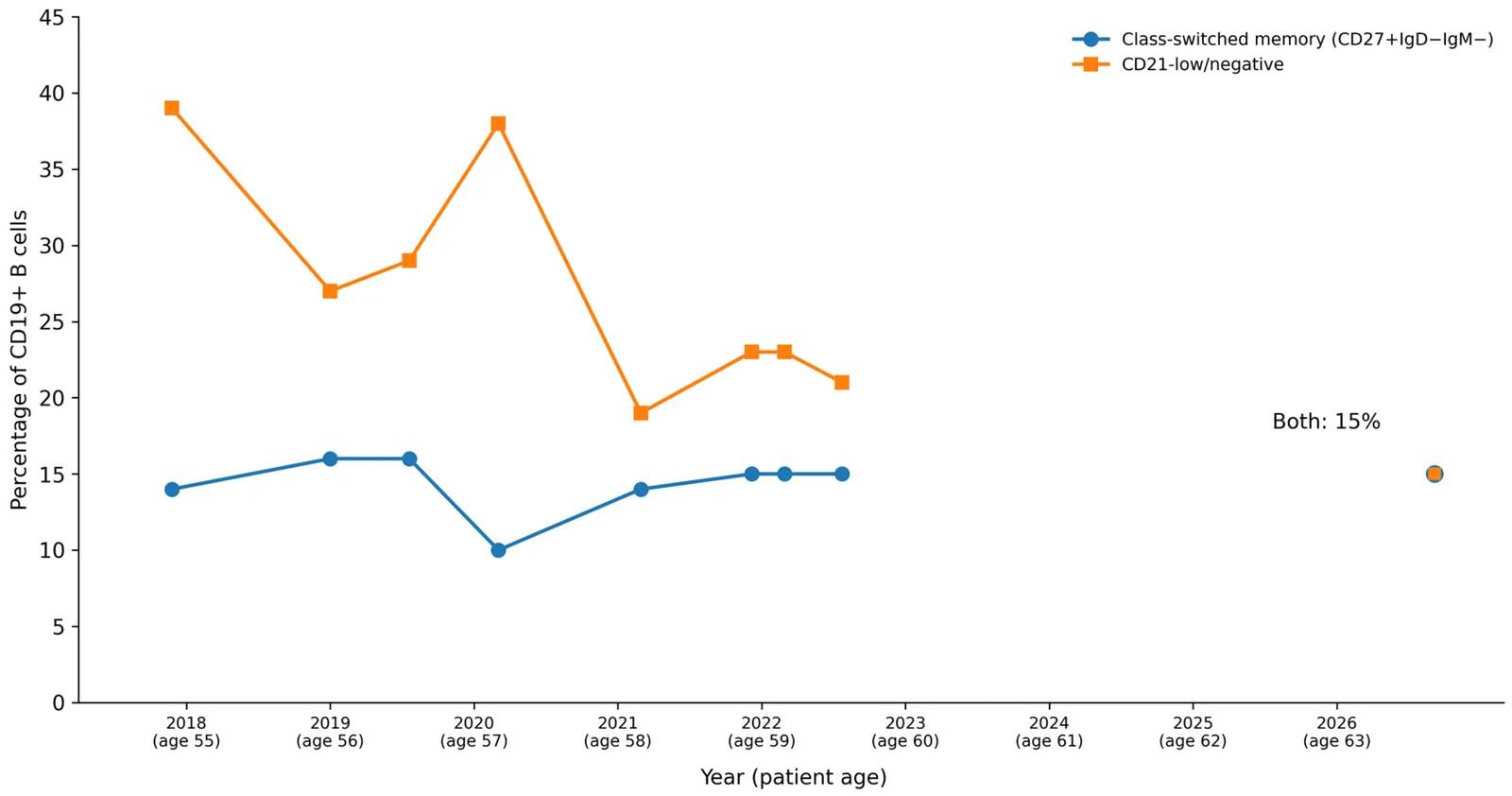
Class-switched memory and CD21-low/negative B-cell proportions, November 2017–September 2026. Values are expressed as percentages of CD19+ B cells. No line is drawn across the July 2022–September 2026 sampling interval. Both populations represented 15% of B cells in September 2026.

**Figure 4:**
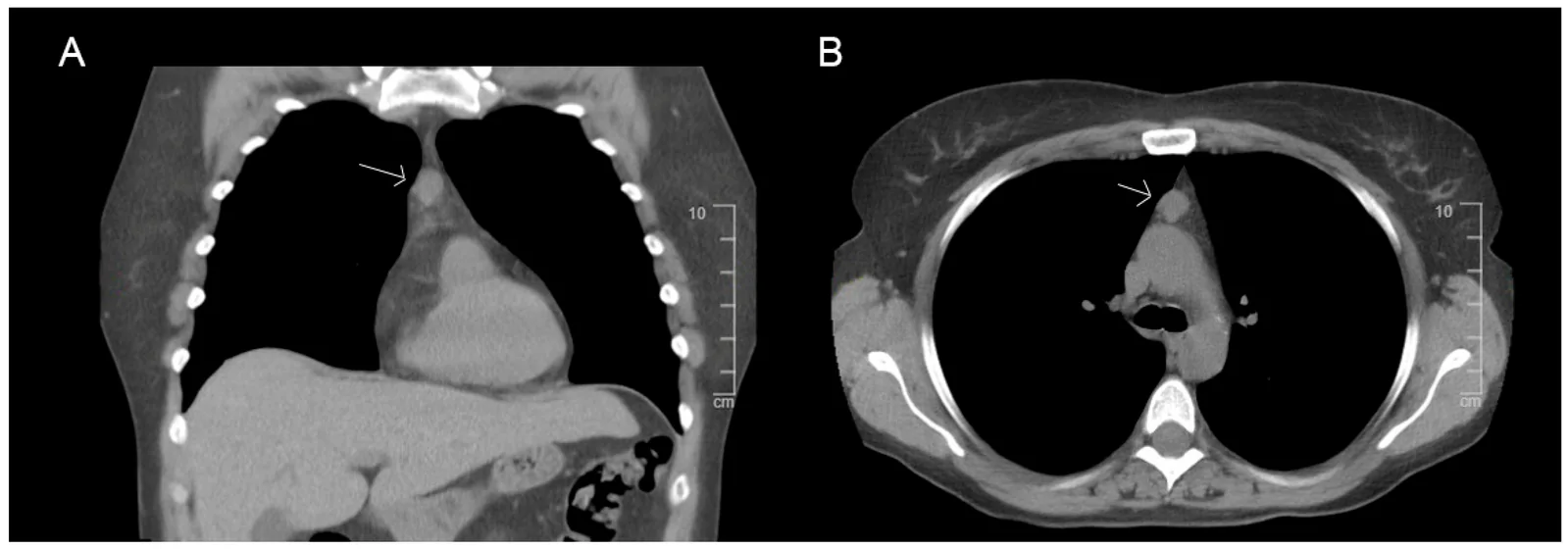
Chest computed tomography at the time of thymoma diagnosis in 2010. Coronal (**A**) and axial (**B**) images show an anterior mediastinal lesion (white arrows).

**Figure 5:**
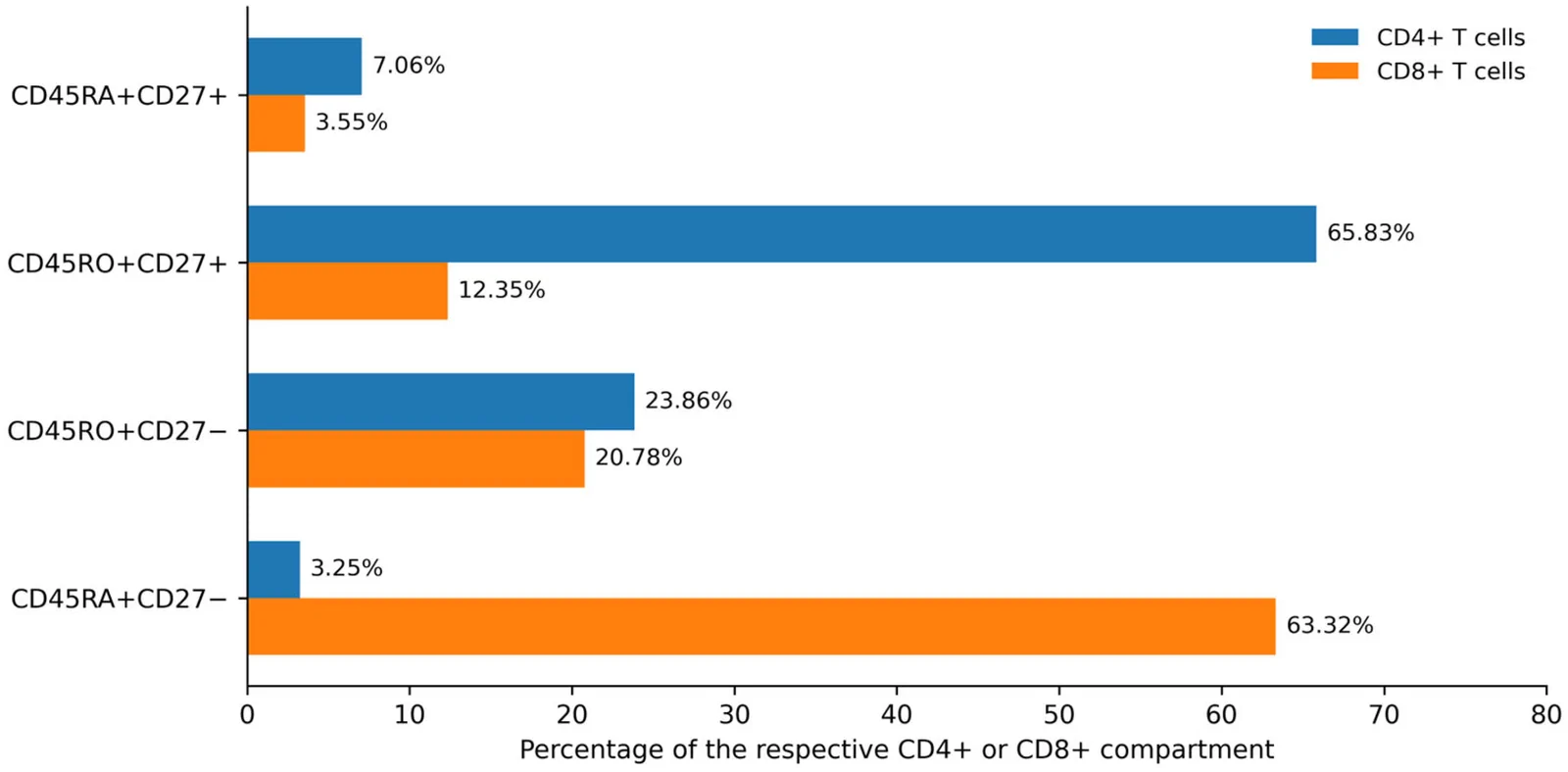
T-cell differentiation in September 2026. Bars show percentages within CD4+ or CD8+ cells, defined by the reported CD45RA/CD45RO and CD27 combinations. Subsets are labeled by these marker combinations rather than by a CCR7-based classification.

**Table 1:** Clinical, imaging, and immunological milestones.

Age (Years)	Observation	Evidence/Outcome
33	Giardiasis, marked diarrhea, and 16 kg weight loss	IgG < 148, IgA < 25.9, and IgM < 19 mg/dL; immunoglobulin replacement initiated. Deficiency persisted after infection resolution.
35–43	Recurrent gastrointestinal symptoms; subsequent resolution	Negative later stool cultures/parasitology; chronic loose stools resolved by 2006.
42–46	Atrophic gastritis, intestinal metaplasia and H. pylori/capsule endoscopy	Three H. pylori eradication regimens; 2009 capsule study normal.
44	HRCT described as normal	No detectable thymic mass or bronchiectasis in the clinical record.
47	Anterior mediastinal lesion; preoperative immunophenotype	CT on 18 March; 25 March CD19 658/µL (21.5%), CD4 1129/µL, CD8 797/µL, NK 211/µL.
	Biopsy, resection and radiotherapy	thymoma, type B2; AB resection; stage II as recorded. Thymectomy was performed and radiotherapy started.
53–63	Serial laboratory B-cell counts	20 measurements: 236–667/µL; median 435.5/µL.
	B-cell phenotyping	CD19 599/µL (21.85%); κ/λ expression 59%/41%; switched memory 15%; CD21-low 15%.
	T-cell differentiation and persistent humoral abnormality	CD4 997/µL; CD8 749/µL; NK 353/µL; reduced naïve fractions; phenotypic recent thymic emigrants 2.7% of CD4; IgA < 2 and IgM < 11 mg/dL.

**Table 2:** Longitudinal class-switched memory and CD21-low/negative B-cell measurements. B denotes CD19+ B cells. Comparisons between time points are limited by differences in sampling intervals and possible changes in panels and gating.

Age (Years)	CD27+IgD−IgM− (% of B)	CD21-Low/Negative (% of B)
54	14	39
55	16	27
56	16	29
57	10	38
58	14	19
58	15	23
59	15	23
63	15	15

**Table 3:** Lymphocyte immunophenotype and serum immunoglobulins, 10 September 2026.

Compartment	Population/Analyte	Result	Laboratory Interval/Note
Lymphocyte counts	CD3+ T cells	1766 cells/µL	700–2100
	CD4+ T cells	997 cells/µL	300–1400
	CD8+ T cells	749 cells/µL	200–900
	CD4/CD8	1.33	1.0–3.6
	CD19+ B cells	599 cells/µL; 21.85%	100–500; 6–19%
	NK cells	353 cells/µL	90–600
B cells (% of B)	Light chains κ/λ	59%/41%	Polyclonal
	CD27+ memory ^1^	46%	15–35%
	CD27+IgD−IgM− switched memory	15%	8–23%
	CD21-low/negative	15%	0–10%
	CD38+IgM+ (reported transitional) ^2^	2.5%	0.9–6.3%
	CD38+IgM− (reported plasmablasts) ^2^	0%	0–5%
CD4 subsets (% of CD4) ^3^	CD45RA+CD27+	7.06%	Reduced; numeric interval unavailable
	CD45RA+CD27−	3.25%	Not reported
	CD45RO+CD27+	65.83%	Not reported
	CD45RO+CD27−	23.86%	Not reported
CD8 subsets (% of CD8) ^3^	CD45RA+CD27+	3.55%	Reduced; numeric interval unavailable
	CD45RA+CD27−	63.32%	See note 3
	CD45RO+CD27+	12.35%	Not reported
	CD45RO+CD27−	20.78%	Not reported
Recent thymic emigrants	CD3+CD4+CD45RA+CD31+ (% of total CD4)	2.7%	>7% (laboratory reference)
Immunoglobulins	IgG/IgA/IgM	743/<2/<11 mg/dL	600–1700/70–400/40–230

^1^ The numeric report identifies 46% as total CD27+ memory, although the accompanying description uses “pre-switch”; an unswitched fraction cannot be assigned from these data. ^2^ CD38/IgM populations follow the laboratory labels; additional gating details were unavailable, and 0% was within the stated plasmablast reference interval. ^3^ CD45RA/CD45RO and CD27 subsets combine CD197-negative and -positive cells and are not presented as CCR7-defined populations. B-cell subsets are percentages of CD19+ cells; T-cell subsets are percentages of CD4+ or CD8+ cells, as indicated. IgG was measured during replacement therapy.

**Table 4:** Clinical definitions, proposed criteria, and phenotypic classifications of GS.

Source	Definition/Description	B-Cell Requirement	Purpose
ESID 2019 [8]	Thymoma plus serum IgG more than 2 SD below the age-adjusted mean	No threshold	Working definition for probable clinical diagnosis/registry
AAAAI/ACAAI/CIS 2026 [9]	HGG plus thymoma supports a GS diagnosis	No threshold	Clinical practice recommendation
IUIS 2024 update [10]	IEI phenocopy; reduced CD4, increased CD8, “No B cells”, low Ig, infections/immune dysregulation	Characteristic findings, not a mandatory criterion	Classification
Kabir et al. 2022 [16]	Proposal to add reduced/absent B cells to diagnosis	Proposed additional criterion	Expert proposal; no validated consensus
Jia et al. 2026 [17]	Thymoma and HGG used to define GS	Cellular findings supportive	Research case definition
Lim et al. [13]	TAI including classical GS and other immune phenotypes	Varies with phenotype	Broader clinical spectrum

**Table 5:** Selected reports of thymoma and hypogammaglobulinemia with preserved peripheral B cells.

Observation	Temporal Relationship	B-Cell Finding	Additional Context
Manzanell et al. 1985 [19]	Thymoma → HGG, 11 years	Normal B cells and marrow plasma cells	Functional T-helper abnormality
Montella et al. 2003 [20]	Thymoma-associated HGG	A panhypogammaglobulinemic patient had B cells within the study reference range	Cohort evidence that B-cell depletion and HGG were not interchangeable
Hashiro et al. 2024 [21]	Thymoma with HGG	190 B cells/µL reported	Pure red-cell aplasia and subclinical myasthenia gravis
Present patient	Documented HGG → thymoma, 14 years	658/µL before thymectomy; 20 counts in 2016–2026, 236–667/µL; 599/µL on 10 September 2026	Polyclonal B cells; switched memory 15%; altered T differentiation without sustained profound CD4 depletion

## Data Availability

The de-identified data supporting the findings are included in the article and its Supplementary Materials. Access to additional source material is subject to patient confidentiality and institutional data-access requirements.

## Supplementary Materials

The following supporting information can be downloaded at: https://www.mdpi.com/article/10.3390/ijms27219726/s1.

## Abbreviations

The following abbreviations are used in this manuscript:

CVID	common variable immunodeficiency
GS	Good syndrome
HGG	hypogammaglobulinemia
HRCT	high-resolution computed tomography
IEI	inborn error of immunity
IUIS	International Union of Immunological Societies
ESID	European Society for Immunodeficiencies
IgRT	immunoglobulin replacement therapy
NK	natural killer
TAI	thymoma-associated immunodeficiency
