# Supplementary material for: Does Thymoma Plus Hypogammaglobulinemia Define Good Syndrome? A 30-Year Longitudinal Study of a Single Patient with Persistent B-Cell Preservation
Source: Int J Mol Sci. 2026 Oct 29;27(21):9726. doi: 10.3390/ijms27219726 (PMC13763255; doi:10.3390/ijms27219726)
Supplement: Supplementary file 1 [file ijms-27-09726-s001.zip › ijms-4613159-supplementary.pdf]

Supplementary Material

Does Thymoma Plus Hypogammaglobulinemia Define Good Syndrome? A 30-Year Longitudinal Study with Persistent B-Cell Preservation

Daniel Iguasnia Portilla[1], Isabel Castillo Castillo[1], Helena Fernández Cabrera[1], Valeria Franco Figueroa[1], Silvia Romero Chala[1], Irina Sobieschi[1], Hector Cordero [2,3,*], Luis Miguel Fernández Pereira [1,*]

[1] Department Service of Immunology and Genetics, Hospital Universitario San Pedro de Alcántara, Cáceres, Spain.
[2] Immunology Unit, Department of Physiology, Faculty of Veterinary Medicine, Universidad de Extremadura, Cáceres, Spain.
[3] Corresponding author: H.C., cordero@unex.es
* These authors contributed equally as senior authors

Data cutoff: 10 September 2026. All values are from a single patient. Dates refer to clinical observations, not independent participants. Direct identifiers and original patient-labeled source files are not included.

Data sources and curation notes

Data sources were the historical clinical case table (including the pre-thymectomy 2010 observation), legacy and updated longitudinal laboratory exports, the final laboratory report dated 10 September 2026, and the CT image included in the revised manuscript. Blank values remain missing; a less-than sign indicates a laboratory reporting bound rather than zero.

Table S1. Longitudinal CD19+ observations and inclusion status. Values are retained as reported. The August 2013 value is shown for transparency but excluded from analysis.

Date	CD19 cells/µL	CD19 %	Source	Inclusion status
25 Mar 2010	658	21.5	D1	Included; outside summary window
05 Nov 2012	287	16.06	L2	Included; outside summary window
08 Aug 2013	130	22.1	L2	EXCLUDED: discordant legacy absolute value
01 Oct 2015	175	11.31	L2	Included; outside summary window
22 Jan 2016	295	16.2	L2	Included; 2016–2026 summary
08 Jul 2016	351	17.67	L2	Included; 2016–2026 summary
28 Nov 2017	271	17.78	L2	Included; 2016–2026 summary
27 Dec 2018	272	17.63	L2	Included; 2016–2026 summary
12 Jul 2019	370	17.63	L2	Included; 2016–2026 summary
21 Feb 2020	236	19.44	L2	Included; 2016–2026 summary
26 Nov 2020	396	19.95	L2	Included; 2016–2026 summary
18 Feb 2021	422	19.02	L2	Included; 2016–2026 summary
12 May 2021	608	19.72	L2	Included; 2016–2026 summary
25 Nov 2021	399	19.68	L2	Included; 2016–2026 summary
17 Feb 2022	473	20.39	L2	Included; 2016–2026 summary
14 Jul 2022	361	16.72	L2	Included; 2016–2026 summary
15 Jun 2023	449	19.62	L2	Included; 2016–2026 summary
23 May 2024	613	20.89	L2	Included; 2016–2026 summary
05 Dec 2024	574	21.74	L2	Included; 2016–2026 summary
30 Jan 2025	532	22.23	L2	Included; 2016–2026 summary
17 Jul 2025	454	24.69	L2	Included; 2016–2026 summary
29 Jan 2026	512	21.87	L2	Included; 2016–2026 summary
24 Apr 2026	667	20.45	L2	Included; 2016–2026 summary
10 Sep 2026	599	21.85	L2	Included; 2016–2026 summary

Twenty-four source observations are listed: one 2010 historical value plus 23 laboratory-export values. The excluded August 2013 value is not plotted and is not used in any summary. Twenty observations in January 2016–September 2026 yield a median of 435.5 cells/µL (range 236–667). Percentages and absolute counts are retained as reported, not recomputed.

Table S2. Major lymphocyte counts reported as absolute counts (cells/µL) and CD4/CD8 ratio.

Date	CD4	CD8	CD19	NK	CD4/CD8
26 Nov 2020	763	668	396	215	1.14
18 Feb 2021	742	764	422	244	0.97
12 May 2021	1049	1269	608	367	0.83
25 Nov 2021	670	793	399	203	0.84
17 Feb 2022	812	940	473	213	0.86
14 Jul 2022	845	858	361	219	0.98
15 Jun 2023	755	836	449	285	0.90
23 May 2024	990	1007	613	363	0.98
05 Dec 2024	801	812	574	313	0.99
30 Jan 2025	777	866	532	282	0.90
17 Jul 2025	711	546	454	154	1.30
29 Jan 2026	789	642	512	281	1.23
24 Apr 2026	1060	913	667	453	1.16
10 Sep 2026	997	749	599	353	1.33

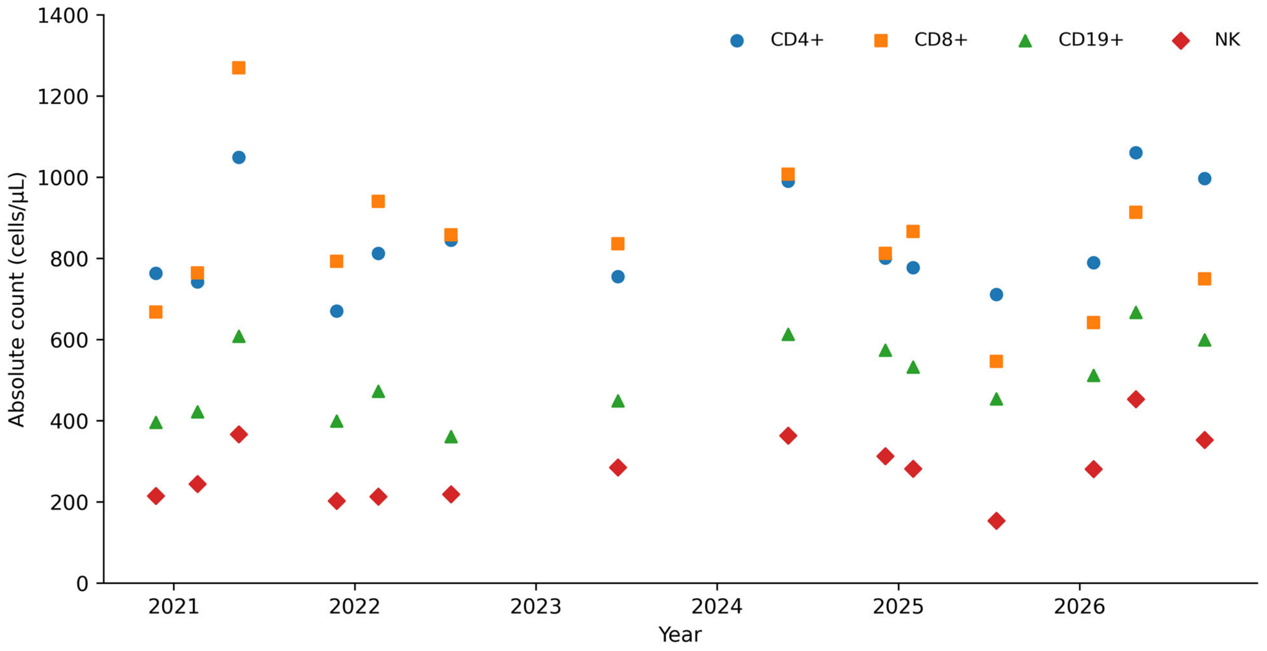

Figure S1. Absolute T-, B- and NK-cell counts. Each symbol is a measured observation. Serial counts are within-patient follow-up data; no interpolation or between-group statistical comparison is implied. Preserved totals do not establish normal cell function.

Table S3. CD38/IgM-defined B-cell populations. Historical and current source-defined populations, expressed as percentages of CD19+ B cells.

Date	CD38+IgM+ (%)	CD38+IgM− (%)
28 Nov 2017	2.7	0
27 Dec 2018	1.7	0
12 Jul 2019	2	0
21 Feb 2020	0.2	0
18 Feb 2021	2.7	0
25 Nov 2021	1.8	0
17 Feb 2022	1.7	0
14 Jul 2022	2.7	0
10 Sep 2026	2.5	0

These are the recorded CD38/IgM-based population labels. They are not assumed to be equivalent to a contemporary CD24-high/CD38-high transitional gate or CD27-high/CD38-high plasmablast gate. In September 2026 the laboratory intervals were 0.9–6.3% and 0–5%, respectively. An explicitly reported 0% does not prove absent marrow plasma cells or a differentiation block.

Table S4. Longitudinal immunoglobulins and reporting limits. Serum immunoglobulin values with the original reporting bounds (mg/dL).

Date	IgG	IgA	IgM
05 Nov 2012	743	<24.3	<16
25 Jan 2013	780	<25.5	<17
08 Aug 2013	799	<25.5	<17
20 Feb 2014	717	<24.5	<15.6
12 Jun 2014	761	<24.9	<15.3
22 Jan 2015	715	<25	<17.3
01 Oct 2015	839	<24.5	<17.8
22 Jan 2016	791	<6.5	<4.47
18 Mar 2016	727	<6.5	<17.9
08 Jul 2016	842	<6.16	<17.3
28 Oct 2016	800	<6.66	<4.73
19 May 2017	813	<6.56	<4.19
30 Nov 2017	813	<2	<10
22 Feb 2018	790	<2	<9
27 Dec 2018	680	<2	<11
22 Jan 2019	749	<2	<11
12 Jul 2019	809	<2	<10
04 Oct 2019	778	<2	<10
21 Feb 2020	746	<2	<10
03 Mar 2020	1022	<2	<10
14 May 2020	823	<5	<5
07 Aug 2020	445	<2	<10
04 Sep 2020	736	<2	<10
26 Nov 2020	781	3	<10
18 Feb 2021	846	<2	<10
12 May 2021	859	<2	<10
10 Jun 2021	866	<2	<10
17 Feb 2022	836	2	<10
14 Jul 2022	—	—	—
11 Aug 2022	844	<2	<10
01 Dec 2022	851	<5	<5
23 Mar 2023	806	2	<10
15 Jun 2023	803	<2	<10
23 May 2024	749	21	<10
05 Dec 2024	752	<2	<10
30 Jan 2025	755	3	<10
17 Jul 2025	683	<2	<10
29 Jan 2026	769	2	<11
24 Apr 2026	737	<2	<10

Date	IgG	IgA	IgM
10 Sep 2026	743	<2	<11

All dates are during immunoglobulin replacement. IgG concentrations are not direct estimates of endogenous synthesis and are not assumed to be troughs. Values preceded by < are left-censored results. Changing numerical reporting bounds do not establish a biological change. IgA 21 mg/dL in May 2024 is retained; the phrase "always undetectable" is therefore not used. The source value "1.022" for IgG in March 2020 uses Spanish thousands notation and is displayed as 1022 mg/dL. The non-numeric source token "(X)" in July 2022 is displayed as missing (—), not zero.
